# Generation of interleukin-13 receptor alpha2 antigen expressing modified vaccinia ankara recombinant virus for potential cancer immunotherapy

**DOI:** 10.1186/2051-1426-2-S3-P58

**Published:** 2014-11-06

**Authors:** Yuki Sato, Ramjay Vatsan, Bharat H Joshi, Syed R Husain, Raj K Puri

**Affiliations:** 1 Division of Cellular and Gene Therapies, Center for Biologics Evaluation and Research, FDA, Bethesda, MD, United States

## 

Genetically modified recombinant poxviruses have shown promise in preclinical models of cancer immunotherapy due to their ability to induce effective cell-mediated immunity against target tumor-associated antigens (TAA). One such vector, recombinant Modified Vaccinia Ankara (MVA), is capable of expressing foreign genes in infected host cells. MVA is replication restricted in most mammalian cells exemplifying a unique safety profile. We have demonstrated that the interleukin-13 receptor α2 (IL-13Rα2) is selectively expressed in various solid tumors but not in normal tissues making it a promising TAA. Prophylactic and therapeutic vaccination with a plasmid vector expressing IL-13Rα2 caused only partial regression of established tumors [1], suggesting that host immune responses against IL-13Rα2 needed further enhancement. Thus, we constructed a recombinant MVA (rMVA-IL13Rα2) expressing both IL-13Rα2 and a green fluorescent protein (GFP) reporter gene. Purified virus titration by immunostaining using anti-vaccinia antibody and anti-IL-13Rα2 antibody confirmed the identity and purity of the recombinant MVA. Western Blot analysis showed the presence of IL-13Rα2 protein (65 kDa). Flow cytometric analysis of IL-13Rα2 negative T98G glioma cells infected with rMVA-IL13Rα2 virus (T98G-IL13Rα2) demonstrated surface expression of IL-13Rα2, indicating the infectivity potential of the recombinant virus. Incubation of T98G-IL13Rα2 cells with varying concentrations (0-100 ng/ml) of IL13-PE (interleukin-13 fused to truncated *Pseudomonas *exotoxin [2] resulted in depletion of GFP^+ ^T98G-IL13Rα2 cells in a concentration-dependent manner. Higher concentrations of IL13-PE (10-1000 ng/ml) also inhibited the protein synthesis in T98G-IL13Rα2 compared to cells infected with control pLW44-MVA. We further observed that IL13-PE treatment of rMVA-IL13Rα2 infected chicken fibroblast, DF-1 cells led to a reduction in virus titer compared to untreated cells. These results indicate that rMVA-IL13Rα2 virus can successfully infect mammalian cells and express IL-13Rα2 in a biologically active form on the cell surface. The immunization studies of rMVA-IL13Rα2 are ongoing in a syngeneic mouse model of metastatic breast carcinoma. Based on *in vitro *results, we expect the rMVA-IL13Rα2 to be a useful agent in tumor immunotherapy as a vaccine alone and in combination with other therapeutic agents to eradicate metastatic tumors.

## References

[B1] Nakashima H Terabe M Husain SR Puri RK A novel combination immunotherapy for cancer by IL-13Ra2-targeted DNA vaccine and immunotoxin in murine tumor models J Immunol 2011 187 4935 4946 10.4049/jimmunol.1102095 22013118 PMC3730529

[B2] Husain SR Puri RK Interleukin-13 receptor-directed cytotoxin for malignant glioma therapy: from bench to bedside J Neuro-Oncol 2003 65 37 48 10.1023/A:1026242432647 14649884

